# Sonochemical-Assisted Biogenic Synthesis of Theophrasite β-Ni(OH)_2_ Nanocluster Using Chia Seeds Extract: Characterization and Anticancer Activity

**DOI:** 10.3390/nano12111919

**Published:** 2022-06-03

**Authors:** Hanaa A. Hassanin, Amel Taha

**Affiliations:** 1 Department of Chemistry, College of Science, King Faisal University, P.O. Box 400, Al-Ahsa 31982, Saudi Arabia; haessa@kfu.edu.sa; 2 Department of Chemistry, Faculty of Science, Ain Shams University, Abbassia, Cairo 11566, Egypt; 3 Department of Chemistry, Faculty of Science and Technology, Al-Neelain University, Khartoum 11121, Sudan

**Keywords:** β-Ni(OH)_2_, chia extract, nanocluster, cytotoxicity, sonochemical

## Abstract

Theophrasite β-Ni(OH)_2_ nanocluster were fabricated via the sonochemical-assisted biogenic method using chia seeds extract as a reducing and stabilizing agent. The optical and morphological feature of the synthesized nanocluster was characterized using UV-Vis, FTIR, FE-SEM-EDS, HR-TEM, DLS, XPS, and XRD analysis. According to FE-SEM and HR-TEM images of the synthesized materials, β-Ni(OH)_2_ nanocluster illustrates the hexagonal particle shape with an average size of 5.8 nm, while the EDS results confirm the high purity of the synthesized nanocluster. Moreover, the XRD pattern of the synthesized materials shows typical peaks that match the reference pattern of the Theophrasite form of β-Ni(OH)_2_ with a hexagonal crystal system. The XPS analysis illustrates that the prepared samples exhibit both Ni^2+^ and Ni^3+^ with the predominance of Ni^2+^ species. Additionally the in-vitro cytotoxic activity of β-Ni(OH)_2_ nanocluster is tested against the MCF7 cell lines (breast cancer cells). The MTT assay results proved that the synthesized β-Ni(OH)_2_ nanocluster has potent cytotoxic activity against breast cancer cell lines (IC_50_: 62.7 μg/mL).

## 1. Introduction 

Nanoscale materials have recently gained great attention in the scientific disciplines due to their unfamiliar physical and chemical properties compared to their bulk components [1,2]. Many potential applications were reported for these materials such as pharmaceutical research, catalysis, fabrication of semiconductors, electronic manufacturing, and recently medical application, especially for antitumor therapy [3,4,5,6,7]. Various nanoparticles are tested for the in-vitro cytotoxicity on a wide range of cancer cell types [8]. The Toxicity effect depends on the physicochemical properties of metal nanoparticles such as structure and crystal morphology [9]. Huang et al. reported that amorphous TiO_2_ nanoparticles have higher reactivity in the generation of reactive oxygen species (ROS) compared to the anatase and rutile analogs. The cubic and octahedron CeO_2_ showed a less toxic response toward RAW264.7 cells than the rod-shaped CeO_2_ [10]. The charge of the nanomaterial surface may also affect the toxicity influence. The positively charged ZnO nanoparticles showed a higher toxic effect against A549 cells than the negatively charged particles [11]. Different positive charges of Fe nanoparticles (Fe_3_O_4_, carbon-coated Fe, and oleic acid-coated Fe_3_O_4_) produced toxic responses in BEL-7402 cells, the worse toxic response was correlated to a higher positive charge [12,13].

For decades, nickel hydroxide was studied and has attracted considerable attention due to its important applications such as photocatalysis [1], oxidation of alcohol as active electrode material, hybrid-super capacitors, and batteries [14]. Furthermore, Ni(OH)_2_ has been reported to induce inflammation in the lungs of rodents [15].The cytotoxicity of (Ni(OH)_2_ nanoparticles was examined in a comparison with (NiO) nanoparticles in the range of 10 to 100 µg/mL toward human bronchoalveolar carcinoma (A549) and human hepatocellular carcinoma (HepG2) cell lines. The results demonstrate that Ni(OH)_2_ was more toxic than NiO nanoparticles [9]. Nickel hydroxide exists in two main crystalline phases (α-Ni(OH)_2_ and β-Ni(OH)_2_). α-Ni(OH)_2_ is stacked randomly along the axis (001) due to the presence of the anions between layers accompanied by water molecules, whereas the β-Ni(OH)_2_ is perfectly stacked as hexagonal crystals. The morphological control for both morphologies of Ni(OH)_2_ can be achieved by synthetic conditions as well as the incorporation of polymers or surfactants [16].

Various techniques have been developed for the production of metal nanoparticles with high energy consumption and longer reaction time strategies such as chemical deposition [17], sol-gel technique [18], co-precipitation [19], green methods [20,21], hydrothermal [22], solvothermal and sonochemical synthesis [23]. The sonochemical-assisted synthesis method has several advantages, such as morphology control, homogeneity of mixing, and decreased product agglomeration [24]. Using the green protocol such as plant extracts, Microorganisms, and enzymes in nanoparticle synthesis is a new approach nowadays. The plant extract shows various benefits including the lack of any toxins and contaminants as well as being environmentally safe [25,26,27]. Recently it has been reported that herbal and medicinal plants were rich in many biologically active components such as flavonoids, amino acids, alkaloids, tannins, terpenoids, saponins, and phenolics which can be responsible for the reduction of metal to metal nanoparticles [27,28,29,30].

*Salvia hispanica* L. is one of the seasonal herbaceous plants that belongs to the Lamiaceae family, commonly known as Chia, it can be found as dark and white small seeds. The chia seeds are well known and valued as high sources of protein, dietary fiber, minerals, polyphenolic compounds, and oils. In addition, Chia is known for high contents of natural antioxidants such as flavonoids, phenolic compounds, caffeic acid, kaempferol, and chlorogenic acid that can be beneficial to human health [31]. These antioxidants can scavenge free radicals, chelate ions, and donate hydrogens [32]. It usually decreases chronic disease risk (cancer and heart attack), and also offers protection against some diseases such as diabetes and Alzheimer’s [29]. Chia seeds extract is also known to have antimicrobial efficiency as reported by G. K. Divyapriya et al. [33]. Accordingly, these chemical compounds in the chia seeds extract can be considered a model candidate for the reduction and stabilization process of nanomaterials. The dark or white chia seed extract has been applied in the synthesis of Ag nanoparticles [34], CuO and NiO nanoparticles [35]. In the present work chia seeds extract is employed to synthesize β-Ni(OH)_2_ nanocluster using the sonochemical assisted biogenic method as a unique, fast, effective, and eco-friendly method. In addition, the in-vitro cytotoxic activity of β-Ni(OH)_2_ nanocluster is examined against the MCF7 cell lines (breast cancer cells).

## 2. Excremental and Methods

### 2.1. Materials

Nickel (II) chloride hexahydrate (NiCl_2_·6H_2_O, 99.9%), and potassium hydroxide (KOH, ≥85.0%) were purchased from Sigma-Aldrich, St. Louis, MO, USA. White chia seeds were collected from the local market in Hufof, eastern province, Saudi Arabia.

### 2.2. Preparation of Chia Extract

4.0 g of chia seeds were crushed, added to deionized water (100 mL), and heated for 15 min at 80 °C then it was double-filtered using a home sieve system and a Whatman filter paper. Finally, the extract was refrigerated at 4 °C for future use.

### 2.3. Synthesis of β-Ni(OH)_2_ Nanocluster

For the synthesis of Ni(OH)_2_ 0.1 M NiCl_2_·6H_2_O was mixed with chia seeds extract with a volumetric ratio (1:1) at room temperature with constant stirring, then the pH was adjusted to 11.0 by dropwise addition of 1.0 M of KOH solution. The pH measurements were carried out using Orion 2 Star (Thermo Fisher Scientific, Waltham, MA, USA) pH meter. The reaction mixture was then subjected to ultrasonication for 30 min using Power-Sonic 405 (Hwashin, Seoul, Korea) with a working frequency of 40 kHz and a maximum input power of up to 350 W. Finally, the synthesized material was rinsed five times with deionized water and allowed to dry at room temperature. 

### 2.4. Characterization of β-Ni(OH)_2_ Nanoparticles

Various analytical techniques are used to investigate the chemical and physical properties of the synthesized β-Ni(OH)_2_. The optical characteristics of the synthesized sample are investigated to confirm the bio-genic synthesis of β-Ni(OH)_2_ by monitoring the absorption spectra of the solution using a Shimadzu UV–vis spectrophotometer (Kyoto, Japan). DLS (Dynamic light scattering) measurements provide further information about the average hydrodynamic particle diameter (d, nm). FT-IR spectroscopy is used to detect different phytochemical functional groups responsible for the production and stabilization of β-Ni(OH)_2_. The FT-IR analysis was recorded using a Cary 630 FT-IR spectrophotometer (South San Francisco, CA, USA). To study the surface morphology of the synthesized β-Ni(OH)_2_, the FE-SEM (Field emission-Scanning electron microscopy) is recorded using FEI, QUANTA FEG, 250 high-resolution field emission electron microscope (Thermo Fisher Scientific, Hillsboro, OR, USA), linked with a high-angle dark-field detector and X-ray energy dispersive spectroscopy device (EDS). Furthermore, TEM images were collected using (JEOL-JEM-2100, JEOL, Peabody, MA, USA) transmission electron microscopy at 90 kV acceleration voltage by dispersing the sample in ethanol by sonication for 30 min and placing one drop of the suspension on the carbon-coated copper grid (400 mesh) and allowed to dry at room temperature. The particle size distribution was calculated using ImageJ software. X-ray diffraction spectroscopy (XRD) was carried out using an Empyrean X-ray diffractometer, Malvern, UK (Cu Ka radiation with a wavelength of 1.54° A) to determine the crystalline phase of the synthesized Ni(OH)_2_. The nano-crystallite size was calculated using Debye- Scherrer equation from the width of the XRD peaks. The XPS, X-ray photoelectron spectroscopic analysis is used to study the surface composition and the oxidation state of the synthesized samples. The XPS spectrum was recorded using K-ALPHA (Thermo Fisher Scientific, USA) equipped with a monochromatic X-ray Al K-alpha radiation (10 to 1350 eV), 400 μm spot size at pressure 10^−9^ mbar with full-spectrum pass energy 200 eV and at narrow-spectrum 50 eV.

### 2.5. Anticancer Activity Studies

#### 2.5.1. Cell Culture

A human Caucasian breast cancer cell line (MCF7) was obtained from the American Type Culture Collection (Rockville, MD, USA). The cancer cells were cultured in DMEM (Dulbecco’s Modified Eagle Medium) containing 10% fetal bovine serum (FBS) 100 U/mL of penicillin and streptomycin. The cells were cultivated at 37 °C in a humidified environment with 5% CO_2_.

#### 2.5.2. In-Vitro Cytotoxic Activity β-Ni(OH)_2_ by MTT Assay

MTT, 3-[4,5-dimethyl-2-thiazolyl)-2,5-diphenyl-2H-tetrazolium bromide assay was applied to assess the cytotoxicity of the bio-synthesized β-Ni(OH)_2_ against the MCF7 cell line. The MTT assay relay on the cleavage of tetrazolium salt by mitochondrial dehydrogenases in live cells [35,36]. Before the MTT assay, the cells were put in 96-well sterilized microplates (5 × 10^4^ cells/well) and kept at 37 °C with DMSO solutions of the test compounds for 48 h in a serum-free medium. The medium culture without the tested compounds was used as a negative control. After incubation, the media in each well was securely removed and replaced with 40 μL of 2.5 mg/mL MTT. Then the samples were incubated for additional 4 h. 200 μL of DMSO was added to solubilize the purple formazan dye crystals. After that, a SpectraMax Paradigm Multi-Mode microplate reader is used to measure the absorbance at 570 nm. The relative cell mortality is the quantification of the mean percentage of dead cells compared to the control sample [37]. All trials were carried out on different days in triplicate. The obtained values are recorded as the mean ± SD. The probit analysis was performed using SPSS software (SPSS Inc., Chicago, IL, USA) to determine IC_50_ values.

## 3. Results and Discussions

### 3.1. Characterization

#### 3.1.1. UV-Vis Spectroscopy

**Figure 1:**
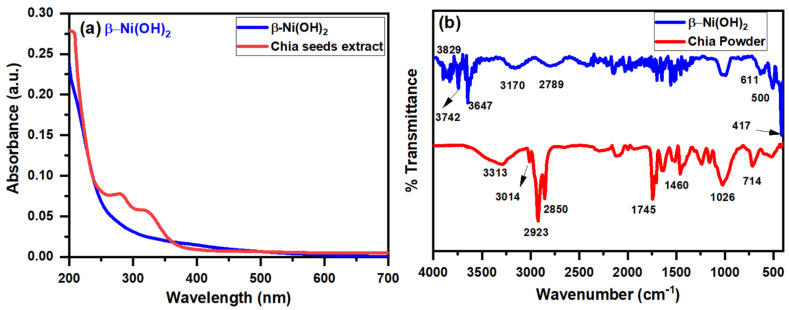
(**a**) UV-Vis spectra and (**b**) FT-IR spectra for chia powder and the biosynthesized β-Ni(OH)_2_.

The optical properties of Ni(OH)_2_-nanocluster and chia seeds extract were evaluated using UV-Vis Spectroscopy. Figure 1a represents the absorption spectra of Ni(OH)_2_ nanocluster and chia seeds extract. The absorption spectrum of chia seeds extract reveals two main peaks in the range of 250–350 nm. The peak centered at 280 nm could be assigned to the xylose and glucose content of chia extract [38] while the peak at 320 could be attributed to the protein content of the chia seeds extract [39]. Due to its weak light-harvesting characteristics, Ni(OH)_2_ has more linear absorption spectra in both the UV and visible regions [40].

#### 3.1.2. FT-IR Spectroscopy

Chia seeds are high in omega-3, polyunsaturated fatty acids, fibers, and proteins, which provide all of the necessary amino acids [41]. FT-IR spectroscopy is performed for chia seeds powder and the synthesized Ni(OH)_2_ nanocluster to identify the functional groups that are responsible for the reduction and stabilization of the prepared nanocluster. Figure 1b shows the FTIR spectra of chia seeds powder and the bio-synthesized Ni(OH)_2_ nanoclusters. The chia protein characteristic bands are observed in the region at 3200–3500 cm^−1^. The broadband at 3300 cm^−1^ is related to the N-H and/or O-H stretching vibration of protein content. The protein amide I groups have characteristic bands in the range 1745–1460 cm^−1^ of C=O bonds. The bands at 2923–2850 cm^−1^ represent the C-H stretching frequency of the methyl and methylene backbones of lipids. The bands at 3014 and 1645 cm^−1^ represent the C=C of linolenic acid and cis-olefins, respectively. The observed band at 714 cm^−1^ is attributed to the bending vibrations of methylene groups in cis-disubstituted olefins [40,41].

The FT-IR spectrum of β-Ni(OH)_2_ shows a characteristic stretching vibrational mod at 3647 cm^−1^ that represents the nonhydrogen bonded (–O–H) groups present in Ni(OH)_2_. Whereas the broad peak at 3170 cm^−1^ is assigned to the stretching vibrations of hydrogen-bonded (–O–H) groups [14]. The bands at 611 and 500 cm^−1^ are related to Ni–O–H bending vibrations. The band at 417 cm^−1^ is related to the stretching vibration of the Ni–O bond [42,43,44]. The residual function groups of chia seed extract are responsible for the appearance of the bands in the range 1702–1451 cm^−1^ [45]. The results confirm the effective biogenic production of β-Ni(OH)_2_ nanocluster.

#### 3.1.3. FE-SEM and EDS Analysis

**Figure 2:**
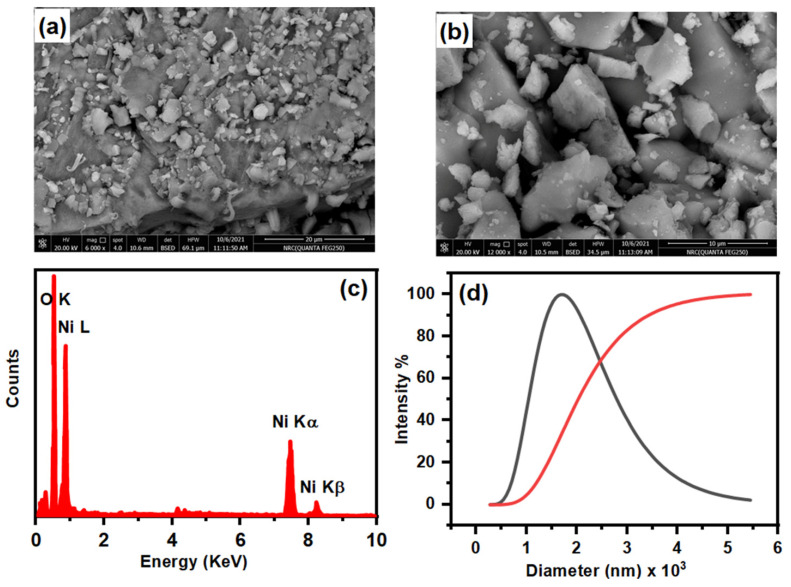
(**a**,**b**) FE-SEM images (**c**) Energy Dispersive Spectroscopy (EDS) and (**d**) Particles size distribution measured by DLS for Ni(OH)_2_-NC.

FE-SEM, field emission-scanning electron microscopy, and energy-dispersive X-ray spectroscopy (EDS) are used to investigate the morphological, structure properties, and the composition of the bio-synthesized β-Ni(OH)_2_ nanocluster. Figure 2a,b show the images of β-Ni(OH)_2_ with different magnification scales. It can be seen that the synthesized materials seem similar to a cluster of nanoparticles. The chia extract is worked as a capping agent for the particles, and they are probably attached by the hydroxyl groups in the molecules, so it gives the appearance of a nanocluster molecule [45,46,47].

Figure 2c shows the energy-dispersive spectroscopy (EDS) microanalysis profile of the β-Ni(OH)_2_ nanocluster. The data illustrates the existence of Ni and O elements only in the nanoparticles which indicates the purity of the synthesized material [48,49].

#### 3.1.4. HR-TEM and DLS Analysis

**Figure 3:**
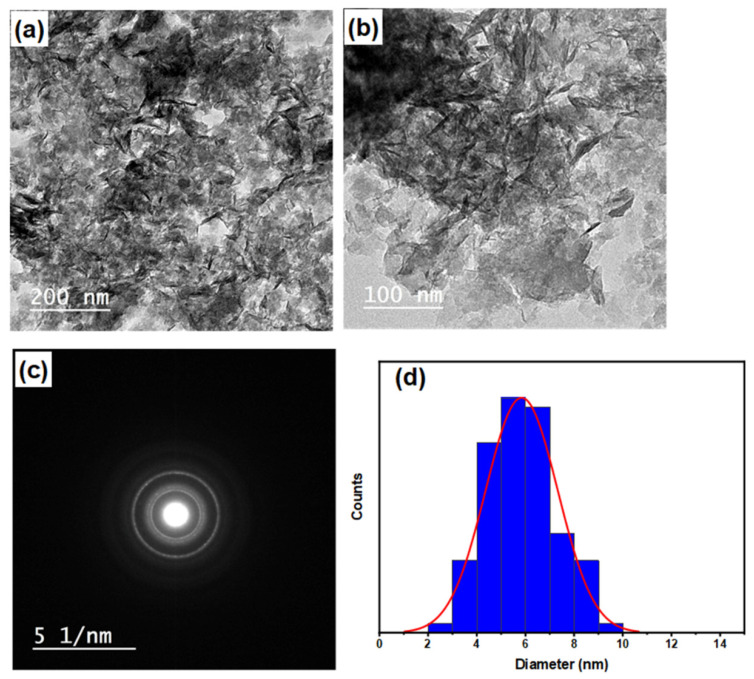
(**a**,**b**) HR-TEM images (**c**) SAED pattern and (**d**) particle size distribution calculated from TEM for β-Ni(OH)_2_ nanoparticles.

Figure 3a,b display HR-TEM images of Ni(OH)_2_ nanocluster. The HR-TEM images show that Ni(OH)_2_ exists as hexagonal thin segments. Figure 3d represents the histogram of particle size distribution calculated using ImageJ software. The estimated average particle size of the nanoparticle from the TEM measurement was 5.8 nm with a standard deviation of 1.48 nm. Figure 2d represents the size distribution for the as-grown nanocluster by the dynamic light scattering (DLS) analysis. The average particle size calculated from DLS is (~2860 nm) with a standard deviation of (~1500 nm). The particle size calculated based on DLS analysis is much greater than that calculated from TEM analysis. TEM measurements reflect only the metal core while the DLS analysis includes the metal core, surface surrounding molecules, and the hydration sphere around the nanoparticle [50]. Another possible explanation for the difference between DLS and TEM particle size measurements is due to the presence of attached layers of different organic molecules (from chia seed extract) to the material nanoparticles, these organic molecules are electron transparent and didn’t appear in TEM [51]. These observations suggest the effective production of Ni(OH)_2_ nanoclusters using chia extract as a capping agent. In the formation of the nanocluster the nickel ions associate with the chia extract molecule as a capping agent. Finally, after completing the nucleation stage, coalescence, Ostwald-ripening, and growth steps Ni(OH)_2_ nanocluster was formed [46]. SAED, the selected area electron diffraction, the pattern of Ni(OH)_2_ nanocluster is shown in Figure 3c, which reveals multi-layered patterns, indicating the polycrystalline nature of the produced Ni(OH)_2_ nanocluster [52].

#### 3.1.5. XRD Analysis

**Figure 4:**
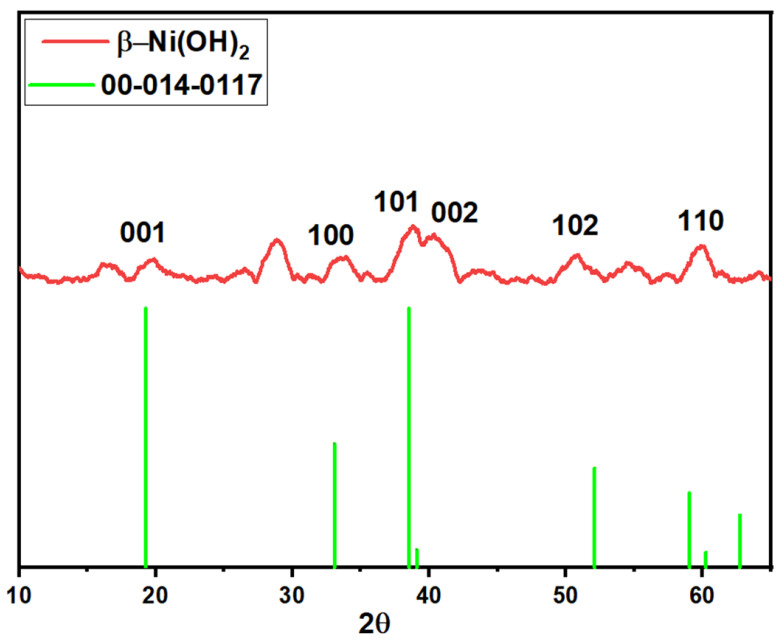
X-Ray Diffraction of Synthesized β-Ni(OH)_2_ nanocluster.

The crystal phase of the prepared Ni(OH)_2_ nanocluster was examined by X-ray diffraction spectroscopy. Figure 4 shows the XRD pattern of the Ni(OH)_2_ nanocluster. The peaks at 2θ values of 19.2°, 33.1°, 38.5°, 39.0°, 52.1°, and 59.5° can be assigned to the diffraction plans (001), (100), (101), (002), (102) and (110), respectively [53,54]. The resulting diffraction pattern matches the reference pattern of the Theophrasite form of β-Ni(OH)_2_ with a hexagonal crystal system according to pdf card number # 014-0117 [55]. The crystallite size of the particles can be estimated by applying the Scherrer Equation (1) [56]:

(1) $$D=\frac{K\lambda }{\beta cos\theta }\;\;\;\;\;\;\;\;\;\;$$

where *D* is the average particle diameter, *K* = 0.9 and is related to the crystallite shape, *λ* refers to the X-ray radiation wavelength and equals 0.15406 Å, *θ* is the peak angle, and *β* is the width at half maximum (FHWM) of the corresponding XRD peak. The average particle size is determined using the Scherrer equation for β-Ni(OH)_2_ nanocluster and found to be 5.5 nm which agrees with the estimated average size from TEM analysis.

#### 3.1.6. X-ray Photoelectron Spectroscopy (XPS) Analysis

**Figure 5:**
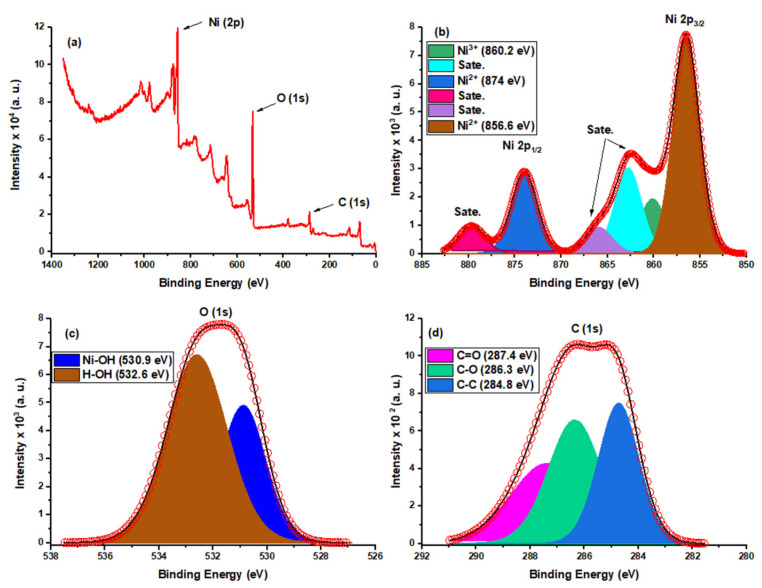
X-ray photoelectron (**a**) Survey scan (**b**) Ni (2p) (**c**) O (1s) and (**d**) C (1s) spectra for the synthesized β-Ni(OH)_2_ nanocluster.

The XPS analysis was performed to study the elemental compositions of the Ni nanoparticle’s surface. Figure 5a shows the survey scan spectrum which indicates the presence of Ni, O, and C elements in the synthesized sample. Figure 5b represents the Ni 2p high-resolution spectrum that contains two spin-orbit doublets of Ni 2p_3/2_ and Ni 2p_1/2_ components at binding energies of 856.6 and 874 eV, respectively with a spin energy separation of 17.4 eV as previously reported for Ni^2+^ in β-Ni(OH)_2_ [53]. Furthermore, the peaks at 862.5 and 879.8 eV are attributed to the shake-up structures of Ni 2p. The de-convolution of the Ni 2p_3/2_ peak results in two peaks located at 856.6 and 860.2 eV, in agreement with the BE values reported for Ni^2+^ and Ni^3+^. The presence of Ni^3+^ on the surface of NP may be due to surface oxidation of β-Ni(OH)_2_ by air [57].

The XPS spectrum of O 1s is shown in Figure 5c. It has a broad peak that The peak at 531.7 eV that can be de-convoluted into two components: the one at 530.9 eV relates to the characteristic oxygen band in (Ni–OH), whereas the peak at 532.6 eV refers to the (–O–H) group of absorbed water molecules (H–OH) on Ni–NP surface, which appears to agree with the previous results of β-Ni(OH)_2_ [58].

The C 1s spectrum of XPS consists of one main peak as shown in Figure 5d. This peak can be de-convoluted to three characteristic peaks at 284.8, 286.3, and 287.4 eV, which are related to C–C, C–O, and C=O, respectively [59]. The presence of C in the β-Ni(OH)_2_ environment is related to the chia extract used in the biogenic synthesis of the material and is proof of the formation of the nanocluster [43]. The binding energy, FWHM, and the atomic percentages of all peaks are listed in Table 1.

### 3.2. Cytotoxicity Evaluation of β-Ni(OH)_2_

**Scheme 1:**
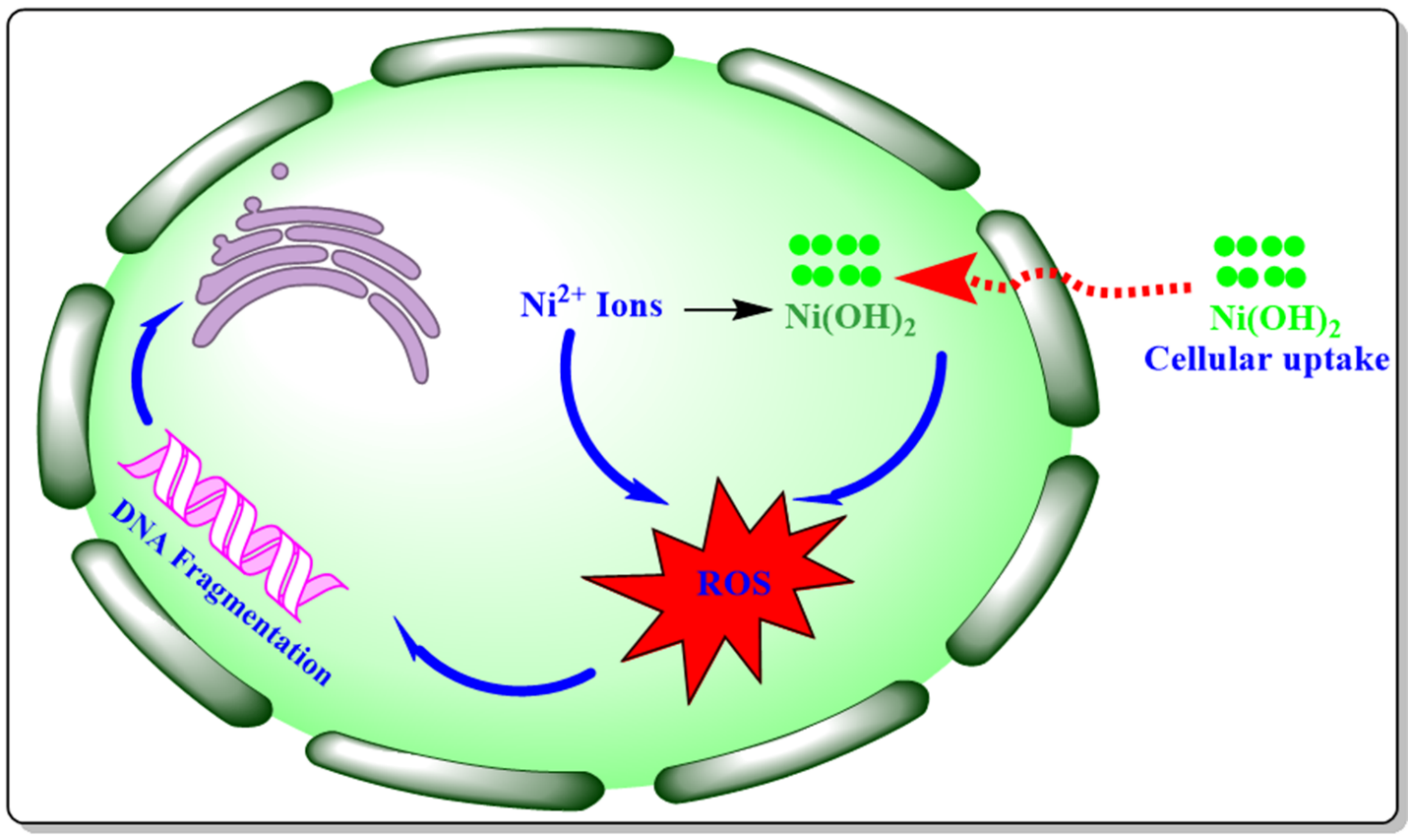
The possible mechanism of the cytotoxic effect of nanomaterials.

Due to the fast developments in the field of nanotechnology, an enormous number of nanomaterials with distinctly shaped nanostructures were produced by different methods [60]. The metal hydroxide and oxide nanostructured materials have a variety of applications due to their distinct properties, such as large surface area, strong reactivity, and small size in the form of various-shaped nanostructures, which are extensively employed as commercial goods such as cosmetics products, food products, medications, textiles, and other applications. Due to their small sizes, nanomaterials may readily penetrate living cells and influence different human organs. Scheme 1 displays the possible mechanism of the cytotoxic effect of nanomaterials as previously reported in the literature [13,61]. The produced nanoparticles may trigger cell death by interacting with the cells, altering the balance between the oxidants and reductants, and generating reactive oxygen species (ROS). The produced ROS species increase the cytosolic Ca^2+^ concentration or trigger the translocation of transcription factors to the nucleus subsequently assembling pro-inflammatory genes. On the other hand, increasing oxidative stress may stimulate the antioxidant defense system and even lead to cell death [62]. 

**Figure 6:**
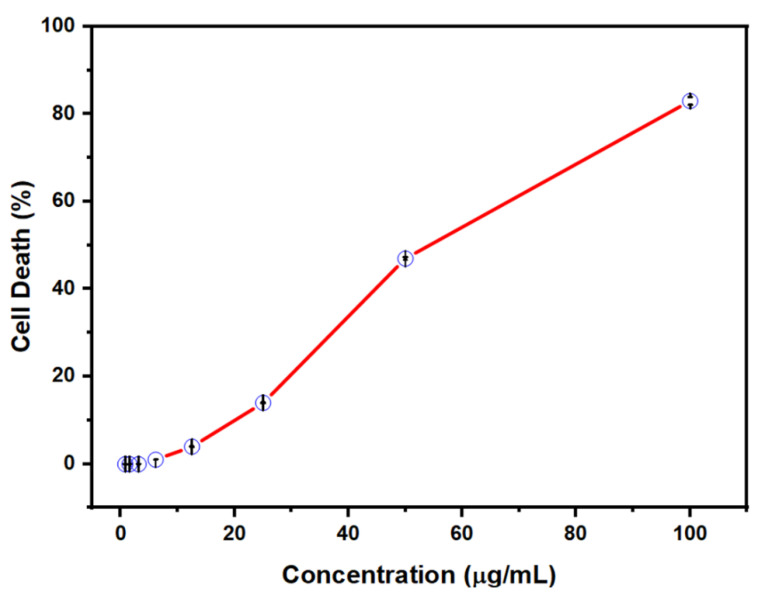
Cell death percentage at different β-Ni(OH)_2_ concentrations.

In this work, the human breast cancer cell line MCF7 was used to evaluate the cytotoxicity effect of the bio-synthesized β-Ni(OH)_2_ using an MTT assay. Figure 6 shows the cell death percentage at different concentrations of β-Ni(OH)_2_ in the range (1.0–100 μg/mL). The cytotoxic effect of β-Ni(OH)_2_ is increasing with increasing the concentration of Ni(OH)_2_ nanocluster indicating a dose-dependent effect on the MCF7 cell line. The IC_50_ value calculated was 62.7 μg/mL and the maximum cell death was found to be 83.2 at 100 ppm.

## 4. Conclusions

In this study, β-Ni(OH)_2_ nanocluster have been synthesized successfully in an aqueous medium by sonication chia seeds extract as a capping and reducing agent. Different analysis techniques were used to study nanoclusters’ physical and chemical properties. According to the results, the synthesized materials are on the nanoscale and have a cluster shape with an average particle size of 5.8 nm as measured by TEM. SEM, EDS, and XRD results indicate that the produced nanoparticles reveal a hexagonal shape of the Theopharasite form of pure β-Ni(OH)_2_. The XPS results show the presence of peaks related to Ni2+, Ni-OH, O-H, C-C, C-O, and C=O confirming the successful biogenic synthesis of Ni(OH)_2_ nanocluster. The in-vitro cytotoxic activity β-Ni(OH)_2_ nanocluster synthesized by using chia seeds extract was tested by using MTT assay. The cytotoxicity of β-Ni(OH)_2_ nanocluster was measured on the MCF7 cell line. The results reveal that β-Ni(OH)_2_ nanocluster were found to be toxic to the studied cell lines with a maximum cell death percentage of 83.2% at 100 ppm of the samples. The number of dead cells indicated the association of the dose toxicity along with a concentration-dependent exposure time.

## Figures and Tables

**Table 1:** Binding energy (eV), FWHM, and atomic percentage for the synthesized β-Ni(OH)_2_.

β-Ni(OH)_2_	Peak (BE)	FWHM (eV)	Atomic%
Ni 2p	857.27	5.52	22.95
O 1s	532.77	4.26	58.95
C 1s	286.51	4.35	18.01
